# Supplementary material for: B3Al4+: A Three-Dimensional Molecular Reuleaux Triangle
Source: Molecules. 2022 Nov 1;27(21):7407. doi: 10.3390/molecules27217407 (PMC9656129; doi:10.3390/molecules27217407)
Supplement: Supplementary file 1 [file molecules-27-07407-s001.zip › molecules-1957122-supplementary.pdf]

SUPPLEMENTARY INFORMATION

$B_3Al_4^+$: A Three-Dimensional Molecular Reuleaux Triangle

Li-Xia Bai,[1] Mesias Orozco-Ic,[2,*] Ximena Zarate,[3] Dage Sundholm,[2] Sudip Pan,[4,*] Jin-Chang Guo,[1,*] and Gabriel Merino.[5,*]

[1] Nanocluster Laboratory Institute of Molecular Science Shanxi University, Taiyuan, 030006, China.

[2] Department of Chemistry, Faculty of Science, University of Helsinki, P.O. Box 55, A. I. Virtasen aukio 1, FIN-00014, Helsinki, Finland.

[3] Instituto de Ciencias Químicas Aplicadas, Facultad de Ingeniería, Universidad Autónoma de Chile, Av. Pedro de Valdivia 425, Santiago, Chile.

[4] Fachbereich Chemie,Philipps-Universitt Marburg Hans-Meerwein-Straße,35043 Marburg, Germany.

[5] Departamento de Física Aplicada, Centro de Investigación y de Estudios Avanzados, Unidad Mérida. Km 6 Antigua Carretera a Progreso. Apdo. Postal 73, Cordemex, 97310, Mérida, Yuc., México.

Table S1. Orbital composition analysis for occupied canonical molecular orbitals (CMOs) of $B_3Al_4^+$ global minimum (C_s, $^1A'$) cluster.

CMO	Al_4 (%)		B_3 (%)	
	s/p	total	s/p	total
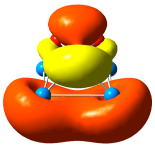<br>HOMO (a')	35.9/31.1	67.0	9.2/21.3	30.5
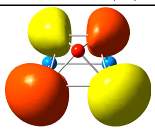<br>HOMO–1 (a")	51.0/19.8	70.8	15.2/12.1	27.3
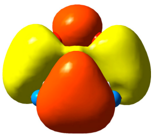<br>HOMO–2 (a')	0.9/43.8	44.7	2.6/50.4	53.0
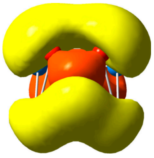<br>HOMO–3 (a')	7.8/25.8	33.7	19.3/44.6	63.9
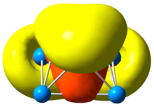<br>HOMO–4 (a')	35.0/11.4	46.4	4.6/46.2	50.9
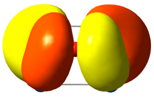<br>HOMO–5 (a")	39.7/9.3	49.0	0.0/49.0	49.0
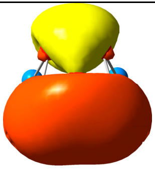<br>HOMO–6 (a')	28.4/6.6	35.0	16.3/45.2	61.5

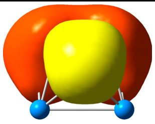 HOMO−7 (a')	37.4/8.5	45.9	11.0/39.5	50.4
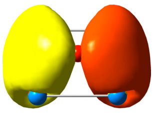 HOMO−8 (a")	25.7/7.2	33.0	21.9/41.7	63.6
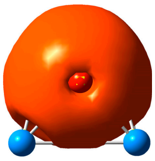 HOMO−9 (a')	7.8/5.8	13.6	52.7/28.8	81.5

Table S2. Orbital composition analysis for occupied canonical molecular orbitals (CMOs) of $B_3Al_4^+$ transition state (C_s, $^1A'$) cluster.

CMO	Al_4 (%)		B_3 (%)	
	s/p	total	s/p	total
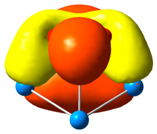 HOMO (a')	45.7/23.8	69.6	11.5/16.7	28.2
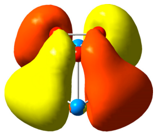 HOMO−1 (a")	3.9/49.5	53.4	6.5/37.8	44.3
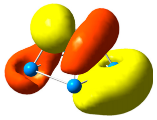 HOMO−2 (a")	37.3/21.3	58.7	9.2/30.2	39.4
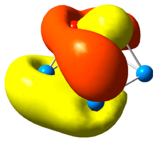 HOMO−3 (a')	24.0/29.4	53.4	12.7/31.4	44.1
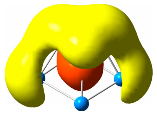 HOMO−4 (a')	20.9/7.6	28.5	10.9/57.8	68.6
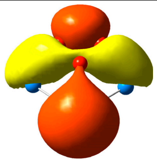 HOMO−5 (a')	38.3/9.9	48.2	0.2/49.6	49.8
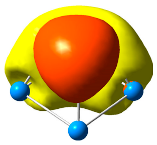 HOMO−6 (a')	30.9/6.8	37.8	11.8/46.7	58.5

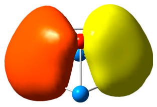 HOMO−7 (a'')	25.5/7.2	32.7	34.4/39.5	64.0
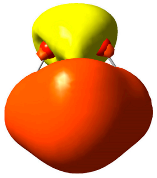 HOMO−8 (a')	35.0/8.4	43.4	12.9/40.1	52.9
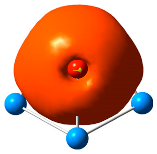 HOMO−9 (a')	7.8/5.8	13.6	52.6/28.8	81.5

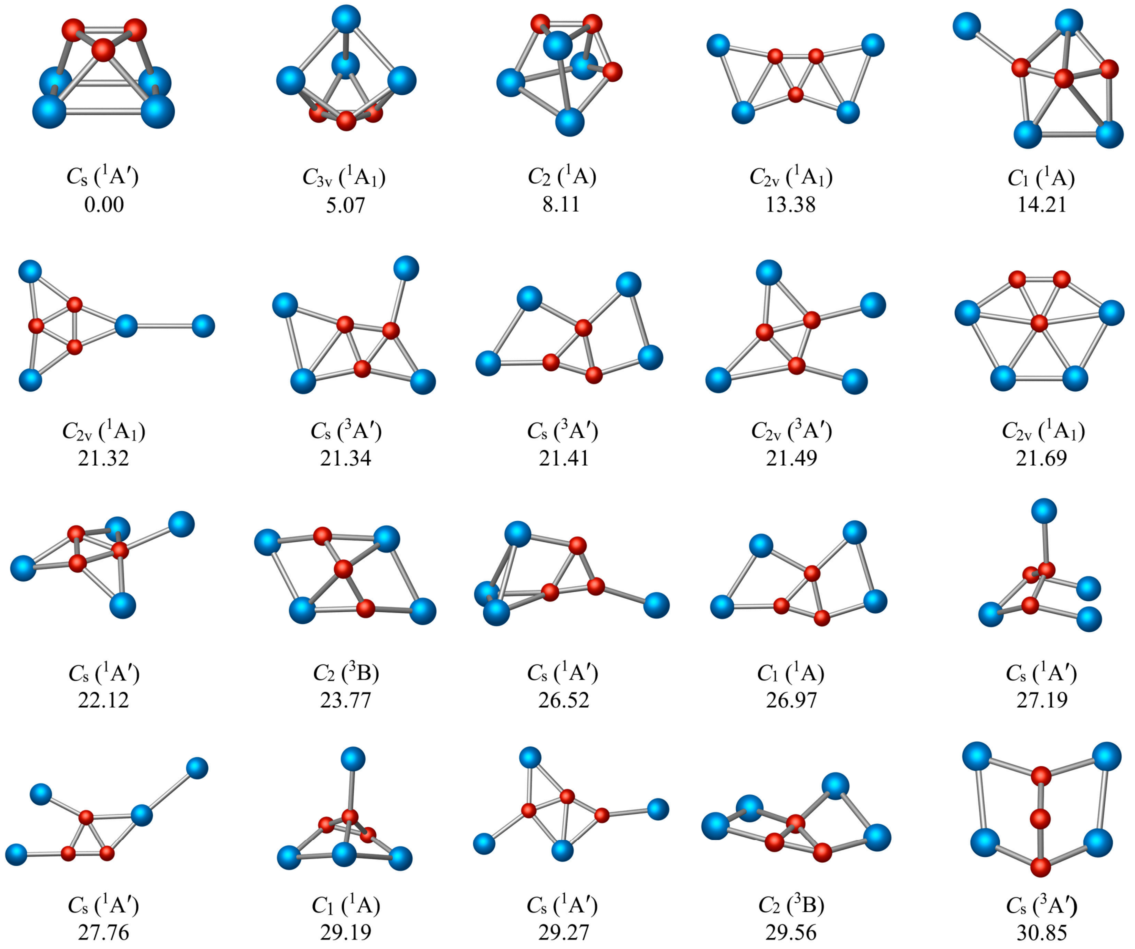

Figure S1. Twenty low-lying isomers of $B_3Al_4^+$. Relative energies are computed at the CCSD(T)/def2-TZVP//PBE0/def2-TZVP level (in kcal mol^{-1}).

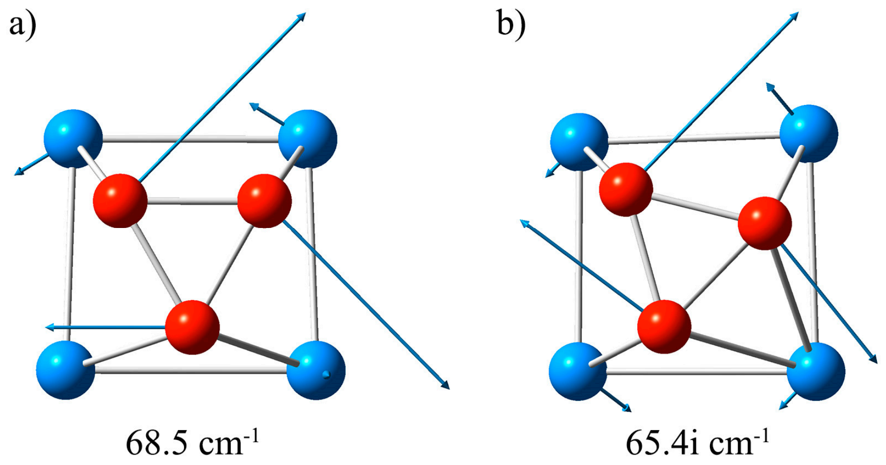

Figure S2. Displacement vectors of **a**) soft vibrational mode of the global minimum and **b**) the imaginary frequency of the transition state of $B_3Al_4^+$.

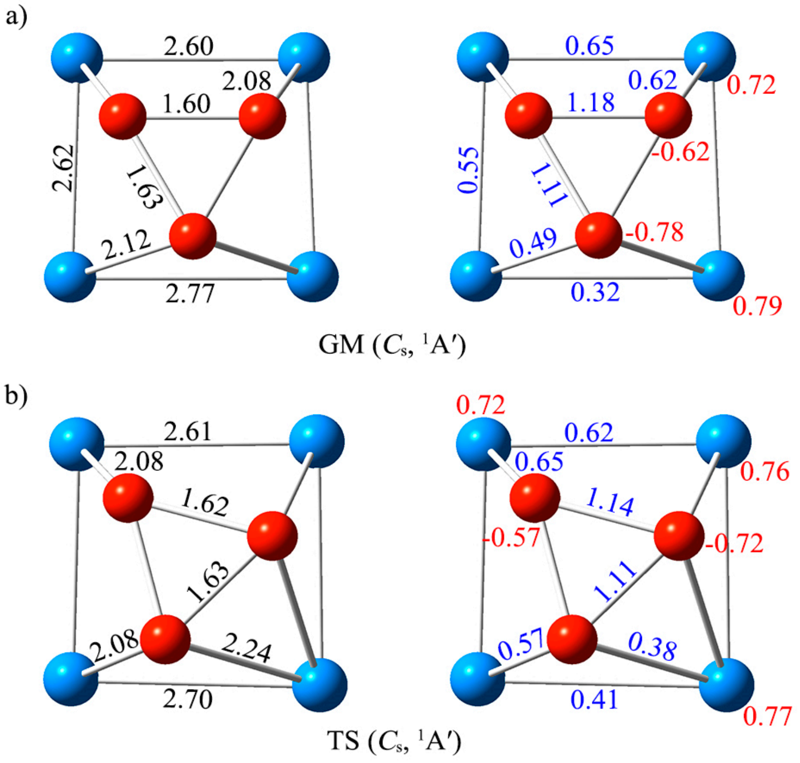

Figure S3. Computed bond distances (black color), Wiberg bond indices (WBIs; blue color) and natural atomic charges (in |e|; red color) for both the global minimum and the transition state structures of $B_3Al_4^+$.

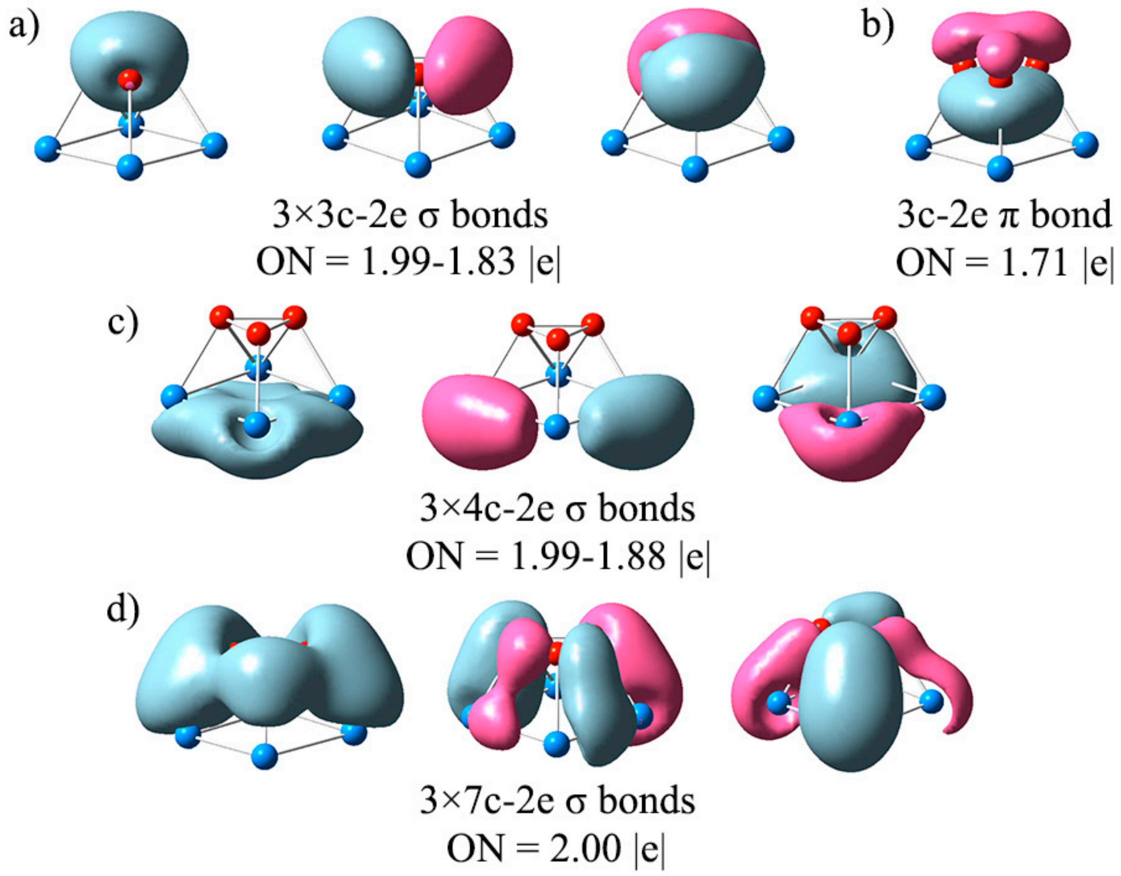

Figure S4. Bonding pattern, according to AdNDP analysis, in the transition state related to the rotation of the B_3 fragment in $B_3Al_4^+$. Occupation numbers (ONs) are shown.

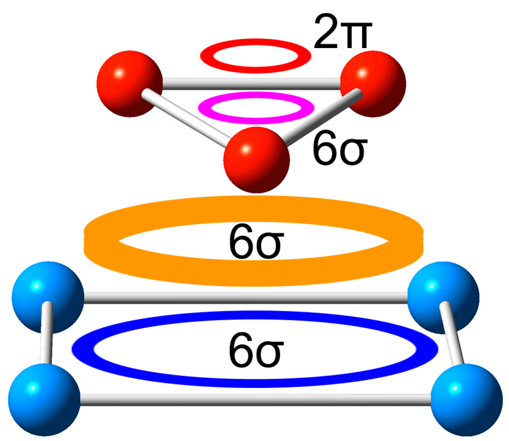

Figure S5. Bonding model for $B_3Al_4^+$.

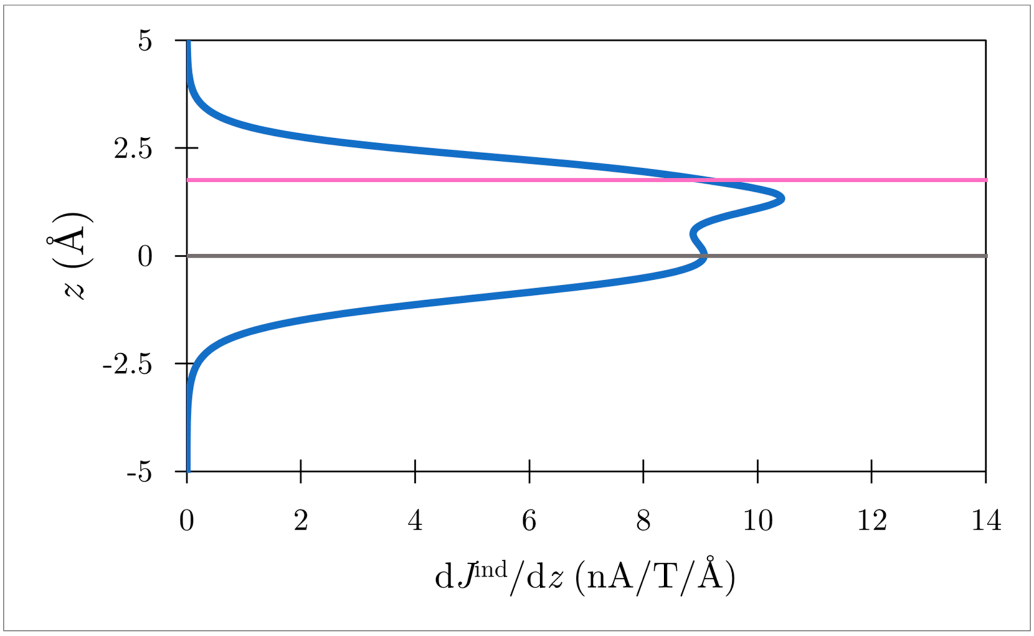

Figure S6. The vertical ring-current profile along the *z*-axis computed at the BHandHLYP/def2-TZVP level. The horizontal pink and gray lines correspond to the *z* values where the plane intersects the B-B and Al-Al bonds, respectively. Integrating the area under the dJ^{ind}/dz curve (in blue) yields the ring-current strength.

Cartesian coordinates at the PBE0/def2-TZVP level

Global Minimum

$B_3Al_4^+$

Al	-1.25489400	0.63262100	1.30092700
Al	0.67957200	-1.12963000	1.38492100
Al	-1.25489400	0.63262100	-1.30092700
Al	0.67957200	-1.12963000	-1.38492100
B	0.67957200	1.21159400	0.80231200
B	0.67957200	1.21159400	-0.80231200
B	1.63252800	0.16125800	0.00000000

Transition State

$B_3Al_4^+$

Al	-1.61037500	-1.06640200	0.00000000
Al	0.07667300	-0.38500100	1.85829700
Al	0.07667300	-0.38500100	-1.85829700
Al	1.87924500	0.37238500	0.00000000
B	-1.25110800	0.97871600	0.00000000
B	0.07667300	1.41386700	-0.81382900
B	0.07667300	1.41386700	0.81382900
